# Quantifying placebo and trial participation effects on cognitive outcome measures in aging dogs

**DOI:** 10.1007/s11357-025-01822-3

**Published:** 2025-08-11

**Authors:** Katherine E. Simon, Katharine Russell, Alejandra Mondino, Chin-Chieh Yang, Beth C. Case, Zachary Anderson, Christine Whitley, Emily Griffith, Margaret E. Gruen, Natasha J. Olby

**Affiliations:** 1 Department of Clinical Sciences, College of Veterinary Medicine, North Carolina State University, Raleigh, NC USA; 2 Department of Statistics, North Carolina State University, Raleigh, NC USA

**Keywords:** Placebo effect, Trial participation effect, Canine cognitive dysfunction, Canine gerontology, Trial design

## Abstract

The placebo effect, or the positive effects observed after an inert treatment which result from patients’ expectations for the therapy, is well documented in human medicine. However, in veterinary medicine, where owner’s expectations serve as a proxy for their pets, it remains underexplored, particularly for elderly dogs with cognitive decline. To address this gap, we examined 21 dogs (mean age: 12.85 years, SD: 1.46) from a placebo group in a randomized controlled trial (RCT) and compared their results to 17 dogs (mean age: 13.24 years, SD:1.56) from an observational, longitudinal study to distinguish placebo effect from trial participation effect. Both populations had statistically comparable baseline data. Cognitive changes were evaluated with two remotely administered owner questionnaires (Canine Cognitive Dysfunction Scale (CCDR) and Canine Dementia Scale (CADES)) and three in-house cognitive assessments (Cylinder Task, Detour and Sustained Gaze). We hypothesized that placebo effect would be greater than trial participation effect, particularly in owner-reported measures. Matched pairs *T*-tests and effect size calculations (Hedge’s *g*) were used to calculate changes across 6 months. A strong (*g* = 0.76), significant (*p* = 0.021) improvement in CADES was observed in the placebo cohort after 6 months, while no changes were detected with CCDR. Conversely, the observational cohort showed a small (*g* = 0.35) and significant (*p* = 0.03) deterioration on CCDR, and no change in CADES. No significant changes were noted on the in-house cognitive assessments in either cohort. We conclude that study context influences remotely delivered owner assessments and CCDR is more robust against caregiver placebo effect in RCTs than CADES.

**Supplementary Information:**

The online version contains supplementary material available at 10.1007/s11357-025-01822-3.

## Introduction

The placebo effect is a phenomenon observed across both human and veterinary medicine. The concept first appeared in the eighteenth century medicine, where an inert substance was used to “satisfy the patient’s demand and his expectations” [1]. Over time, ethical concerns (i.e., treating an individual with a non-treatment) shifted the use of placebos from clinical practice to the research and clinical trial community [2, 3]. Placebos allow patients to experience the natural course of disease along with the psychological and physical aspects of an intervention, but without the active treatment being evaluated [4]. Therefore, they serve as a valuable comparison against a novel therapeutic. This comparison has acted as a vital tool to evaluate the true efficacy of new treatments and remains the gold standard method of control in randomized control trials (RCTs) [5, 6].

The widespread implementation of placebos in RCTs has facilitated a more in-depth exploration of the complex mechanisms underlying the placebo effect. The experience on either a placebo or active treatment is heavily influenced by an individual’s expectations and the contextual framing of the intervention. However, Finniss et al. describes several additional mechanisms which contribute to an observed response that are both psychological/neurobiological (expectancy, conditioning, learning, differential neurotransmitter, or neuromodulatory mechanisms) and non-psychological (natural course of a disease, regression to the mean, response bias, and concurrent treatments) [7]. Moreover, disease conditions and outcome measures may be differentially susceptible to placebo effects [8]. For example, placebo mechanisms in patients with Parkinson’s Disease include activation of dopamine pathways, whereas patients with Alzheimer’s disease (AD) have been shown to have increased connectivity of the prefrontal lobes when treated with a placebo [9, 10]. The size of the placebo effect is well documented across many conditions in human medicine [11–13]. In the case of AD and dementia, placebo effect is influenced by the patient’s baseline cognitive status and disease progression, both of which vary individually [14]. It has also been shown that a contributing factor in populations of elderly patients is the “trial participation effect” [15, 16]. Specifically, improvement is observed due to increased access to medical care/personnel, increased attention to health/routine, or increased observation (i.e., Hawthorne effect) [17, 18]. Therefore, the complexity and varied mechanisms of the placebo effect must be considered when designing clinical trials and interpreting results, particularly in geriatric and cognitively impaired populations.

Dogs with Canine Cognitive Dysfunction Syndrome (CCDS) serve as a valuable pre-clinical model for AD, exhibiting similar neuropathology and behavioral changes [19–22]. However, the placebo effect has not yet been examined in trials of senior dogs with cognitive decline, making it difficult to accurately interpret therapeutic efficacy in CCDS studies. In veterinary medicine, clinical trials often have smaller sample sizes, are seldomly replicated and may operate under lower standard of evidence compared to human trials. These limitations, especially when coupled with lack of control groups or adequate blinding, can exacerbate the contributions of placebo effect to observed outcomes. This is of particular concern with the use of subjective owner-reported outcomes where the owner’s expectations serve as a proxy for their pets [23]. This caregiver placebo response has been reported in studies of canine osteoarthritis, epilepsy, and behavioral syndromes [24–26]. These studies highlight the need to manage placebo effect in veterinary populations, particularly given the growing use of veterinary clinical trials as preclinical studies for human therapeutic development [27].

The placebo response also contributes to various elements of trial design such as randomization, end point selection, blinding, and patient population selection [28]. A crucial part of patient selection is sample size calculation, which is guided by a power analysis. These calculations are dependent on knowledge of the trajectory of the primary outcome with standard management, standard deviation within the population, and anticipated treatment difference (also known as effect size) [28, 29]. However, the limited literature on longitudinal performance of cognitive outcome measures, along with the lack of understanding of placebo effect in dogs with CCDS, makes this variance and effect size difficult to predict. Therefore, it is imperative to establish data which measures these effects to ensure the rigor, validity, and interpretability of future CCDS clinical trials.

Elderly companion dogs from the placebo group of a randomized control trial (RCT) [30] were evaluated across 6 months with five cognitive outcomes: two validated cognitive surveys (Canine Dementia Scale (CADES) and the Canine Cognitive Dysfunction Rating (CCDR)) [31, 32] and three validated in-house cognitive assessments (cylinder tasks (inhibitory control and detour task for executive function) and sustained gaze (for focus and attention)) [33–35]. We hypothesized that duration of the placebo effect in our canine trial would reflect patterns observed in human dementia trials, where the placebo effect typically lasts up to 12 weeks [14]. Accordingly, we predicted that the placebo effect in senior dogs would be present at 3 months, but wane by 6 months. We also hypothesized that in-house cognitive assessments would be less susceptible to placebo effect, given they are more objective measures than the owner assessments. However, we predicted that participation in a study, irrespective of treatment, would yield measurable improvements across all outcome measures. Therefore, we then compared effect sizes of our RCT placebo population to a population of senior animals who underwent similar testing as part of an observational, longitudinal cohort study. This comparison allowed us to distinguish the benefits of participating in a clinical study, which while meaningful, were expected to be less pronounced than the effects associated with the expectation of an intervention (i.e., the placebo effect). Together, these analyses provide a framework for understanding the magnitude and duration of placebo effect and participation effect on owner and in-house cognitive assessments. This will inform the design, analysis, and interpretation of future canine clinical trials.

## Methods

Study protocols underwent review and approval by the North Carolina State University Institutional Animal Use and Care Committee (IACUC # 21–303-O and 21–376-O). All procedures were performed in accordance with these approved protocols and institutional guidelines. Owners of the dogs who participated in these studies reviewed and signed an informed consent form. Institutional review board (IRB) approval was not sought because all collected data pertained to dogs, and as such, the work was categorized as “Non-Human Subject Research.”

### Subjects

Dogs were included from two populations: the placebo group in a recently conducted RCT; and dogs from an ongoing, observational, longitudinal study of the neurological changes of aging. Dogs from both studies were selected using the same inclusion criteria given baseline cognitive function is imperative in evaluating the placebo effect in dementia patients [14]. Dogs were required to be at least mildly cognitively impaired (as indicated by a CADES questionnaire score of 8 or greater [31]) and aged greater than 10 years. Baseline (month 0), 1-month, 3-month, and 6-month outcomes were collected throughout the RCT and used in this analysis. As a part of the RCT, dogs received daily capsules designed to mimic the active treatment in appearance and dosing regimen. The number of capsules administered per day was weight-adjusted and matched the active treatment group. Owners were informed that they were participating in a double-blinded study which would determine the effects of a supplement on the cognition and mobility of their dog. Owners were provided with a dosing calendar to document daily administration and enhance compliance. Participants in the longitudinal, observational study are evaluated on a semiannual basis. Therefore, their baseline (first complete visit with cognitive assessments) and visit two (6 ± 1 month after baseline) data were included. Two dogs participated in both the RCT and the longitudinal cohort study. For these dogs, data from the trial which they participated in first were used ensuring the baseline data from each dog included in this analysis was their (and their owner’s) first attempt at each cognitive assessment.

All dogs were required to have sufficient vision and mobility to perform the in-house cognitive assessments in either study at time of recruitment. All dogs were assessed via physical examination at every visit and laboratory testing was performed at baseline and 6-month visits (complete blood count, serum biochemistry, and urinalysis) to document comorbidities (such as chronic kidney disease). Dogs with severe, unstable systemic disease, which could impact clinical course over the subsequent 6 months (e.g., hyperadrenocorticism, diabetes mellitus, neoplasia), were excluded. Development of a comorbidity over the course of the study did not result in immediate exclusion; however, if the owner felt their dog could no longer complete study visits, they could elect to withdraw.

All studies were conducted at NC State University College of Veterinary Medicine (NCSU CVM). Recruitment and examination of patients was performed by the NC State Canine Neuroaging Program via email, flyers, and social media posts to the NCSU CVM students and staff and surrounding local community.

### Cognitive outcome assessments

Both the CCDR and the CADES are validated owner questionnaires which assess the frequency of behavioral changes associated with canine cognitive impairment (disorientation, house soiling, changes in social interactions and sleep/wake cycles, and increases in anxiety and aggression). The CCDR questionnaire (with scores ranging from 0 to 80) was developed to identify dogs with CCDS (scores ≥ 50) [32]. It also designates dogs who are at risk (scores 40–49). The CADES questionnaire (with scores ranging from 0 to 95) was developed to further differentiate mild, moderate, and severe cognitive impairment. Ranges are designated as follows: normal (0–7), mild (8–23), moderate (24–44), and severe (45–95) [31].

Across both the RCT and the longitudinal, observational study, owners were administered CCDR and CADES online no more than one week prior to their dog’s visit at NCSU CVM. At enrollment into either study, owners were instructed to select one designated person from their household to take all surveys across all visits to maintain consistency. Owners were given detailed instructions at the onset of both studies to ensure the survey was filled out correctly. Survey answers were collected and stored in a REDCap (Research Electronic Data Capture) online database [36, 37].

In previous cross-sectional studies, scores from the CADES questionnaire correlate with performance on three tasks: cylinder (inhibitory control and detour), and sustained gaze performance [38]. Therefore, these three in-house cognitive assessments were included in the analyses. These assessments are performed in the laboratory, by laboratory personnel (away from the owner) which suggests they may be more objective, and robust against a placebo effect. However, longitudinal performance of these tasks has yet to be evaluated. All in-house cognitive tests have been described in detail elsewhere [33–35, 39, 40]. Briefly, on the day of the visit, owners are instructed to feed their dog only half of their morning meal so that their dog is motivated by a food reward. The inhibitory control and detour tasks are both in-house assessments of executive function where the dog must obtain a treat from inside a transparent cylinder. Prior to testing, warm-ups are performed with an opaque covering around the cylinder where the examiner shows the dog a treat, places it in the cylinder and the dog is released so that they may learn to approach and navigate the cylinder to obtain the treat. The dog passes if they can repeat this four times (within five trials) without touching the cylinder. Next, the opaque covering is removed for the testing phase where the dog can see inside the cylinder. Again, the dog is shown a treat and must navigate to the treat without touching the cylinder. This is repeated for eight trials and the percentage of correct (no touch) attempts is calculated. Throughout this inhibitory control phase, the dog’s preferred side is noted. For the detour challenge, the dog’s preferred side is closed off so that the dog must re-learn the task (an exercise in cognitive flexibility) and go to the opposite side to obtain a reward. This is repeated for eight trials and the percentage of correct (no touch) attempts is calculated. The sustained gaze task assesses focus and attention through the time which a dog can maintain eye contact for a treat. Simply, in a room void of distractive noise, objects, and individuals, the examiner calls the dog’s name, offers a verbal cue (“look”) and presents a treat held near their face. The examiner starts a stopwatch at the same time as the verbal cue and times how long the dog maintains eye contact with either the treat or the examiner (with a maximum of 60 s). The time is stopped as soon as the dog breaks eye contact. This is repeated three times, and the mean time is calculated.

### Statistical analysis

Data were summarized for dogs grouped as placebo or observational cohort. Normality of all data was assessed and confirmed via visualization of the residuals and quantile–quantile (QQ) plots. Residual plots were examined for a roughly symmetric, bell-shaped distribution centered around zero. QQ plots were used to compare residuals against a theoretical normal distribution, with close alignment to the reference line indicating normality. Based on these visual assessments, we determined the assumption of normality was appropriate for all data.

Baseline data from both study populations were statistically compared to ensure that both populations had comparable performances across all cognitive assessments at study entry. A two-sample *T*-test was employed for continuous data whereas Fisher’s exact test was employed to compare categorical data (sex) across groups.

Box and whisker plots with 95% confidence intervals were used to visualize individual cognitive assessment performance trajectories for each group over 6 months. These graphs included participants from both cohorts (represented by individual data points). To determine whether individual performance changed compared to study entry, individual baseline scores were compared to each subsequent timepoint (1, 3, or 6 months for the placebo cohort or 6 months for the observational cohort) using a paired *T*-test. The magnitude of these changes (i.e., the magnitude of the placebo effect or the magnitude of trial participation effect) was then quantified by calculating effect size [41]. Hedge’s *g* was chosen to calculate effect size, which is recommended over Cohen’s *d* for small sample sizes [42, 43]. Specifically, Hedge’s *g*_av_ evaluates the magnitude of effect (change) within a consistent group of individuals (as baseline and endpoint scores from the same participant would be inherently correlated) [43].

To directly compare the placebo and observational cohort, individual change in score across each cognitive outcome was determined by subtracting an individual’s baseline scores from their 6-month scores. A two-sample *T*-test and Hedge’s *g* calculation was then used to compare the difference between cohorts. In this instance, because the groups being compared are independent from one another, Hedge’s *g*_s_ was used instead of Hedge’s *g*_av_ [43]. Given the differences between cohorts (namely the different number of follow-up visits), we additionally employed the use of mixed effects models to evaluate the cohort by timepoint interaction. Each model included dog as a random effect, with age, weight, sex, timepoint, cohort, and a cohort by timepoint interaction specified as fixed effects. A significant cohort by timepoint interaction would indicate that the cognitive assessment trajectory differs between the cohorts over time, despite any baseline demographic differences.

For all analyses, *p* values < 0.05 were considered statistically significant. Effect size calculations were performed based on the formulas outlined by Daniël Lakens and can be interpreted in the same manner as Cohen’s *d* effect size, with 0.2 indicating a small effect size, 0.5 indicating medium effect, and 0.8 or greater indicating a large effect size[41]. All remaining statistical analyses and graphical representations were performed using JMP^Ⓡ^ Pro 16.0.0 (SAS Institute Inc., Cary, NC).

Finally, to investigate whether changes were clinically meaningful and comparable to existing literature, a descriptive analysis was performed. Individuals were stratified based on their CADES and CCDR severity at both baseline and 6-month timepoints. Severities were assigned based on survey guidelines [31, 32]. Each dog was further classified as: improved (decreased in severity category), no change (remained in the same severity category) or deteriorated (increased in severity category). Proportions of patients in each severity classification were represented by percentages. This descriptive analysis was performed for both the observational cohort and the RCT placebo cohort.

## Results

### Study population

#### Placebo cohort

This cohort consisted of 21 dogs aged 10 years or older (mean: 12.85, SD: 1.46), with at least mild cognitive impairment as indicated by the CADES assessment (mean: 35 SD: 10.65). Twelve of the placebo participants were male (*n* = 11 neutered, *n* = 1 intact), and nine were female (*n* = 9 spayed). The average weight of the cohort was 22.52 kg (kg) (SD: 9.87). Labrador retrievers were the most represented breed (*n* = 5) followed by golden retriever (*n* = 2), and then there was one of each of the following: beagle, collie, German shepherd, husky, miniature pinscher, pug, and Staffordshire terrier. Seven dogs were listed as mixed breeds. All baseline parameters are provided in Table 1.

**Table 1:** Baseline (0-month) demographic and cognitive outcome performance data by group

	Placebo cohort (*n* = 21)	Observational cohort (*n* = 17)	*p* value
Baseline (0 month) age (years)	Mean: 12.85 SD: 1.46	Mean: 13.24 SD: 1.56	0.44
Sex	Male: *n* = 12 Female: *n* = 9	Male: *n* = 2 Female: *n* = 15	0.07 (2-tailed Fisher’s exact)
Weight (kilograms)	Mean: 22.52 SD: 9.87	Mean: 16.24 SD: 8.38	0.04
Canine Cognitive Dysfunction Rating (CCDR) score	Mean: 38.29 SD: 3.49	Mean: 40.24 SD: 7.68	0.34
Canine Dementia Scale (CADES) score	Mean: 35 SD: 10.65	Mean: 28.94 SD: 16.67	0.21
Cylinder task: inhibitory control performance (% correct)	Mean: 78.75 SD: 21.11	Mean: 65.63 SD: 31.27	0.17
Cylinder task: detour performance (% correct)	Mean: 41.88 SD: 26.98	Mean: 30.85 SD: 32.91	0.32
sustained gaze duration (seconds)	Mean: 24.53 SD: 14.68	Mean: 19.59 SD: 17.65	0.37

*SD*, standard deviation

### Observational study cohort

Seventeen dogs from the longitudinal observational study matched the inclusion criteria of the RCT. These dogs had a statistically comparable baseline age (mean: 13.24 years, SD: 1.56) and a significantly (*p* = 0.04) lower average weight (mean: 16.24 kg SD: 8.38). The cohort had fewer male (*n* = 2 neutered) than the placebo cohort, however the Fisher’s exact test determined this difference was not significant (*p* = 0.07). The mean CADES score of this cohort was 28.94 (SD: 16.67) which was not statistically different from the placebo cohort (*p* = 0.21). This cohort consisted of 5 mixed breed dogs and one of each of the following breeds: basset hound, beagle, border terrier, border collie, Brittany spaniel, cockapoo, corgi, German shorthaired pointer, husky, labrador retriever, pomeranian, Staffordshire terrier. There was no significant difference in baseline cognitive performance on any of the five assessments between the cohorts (Table 1).

### Cognitive performance across 6 months by cohort

#### Placebo cohort

**Fig. 1:**
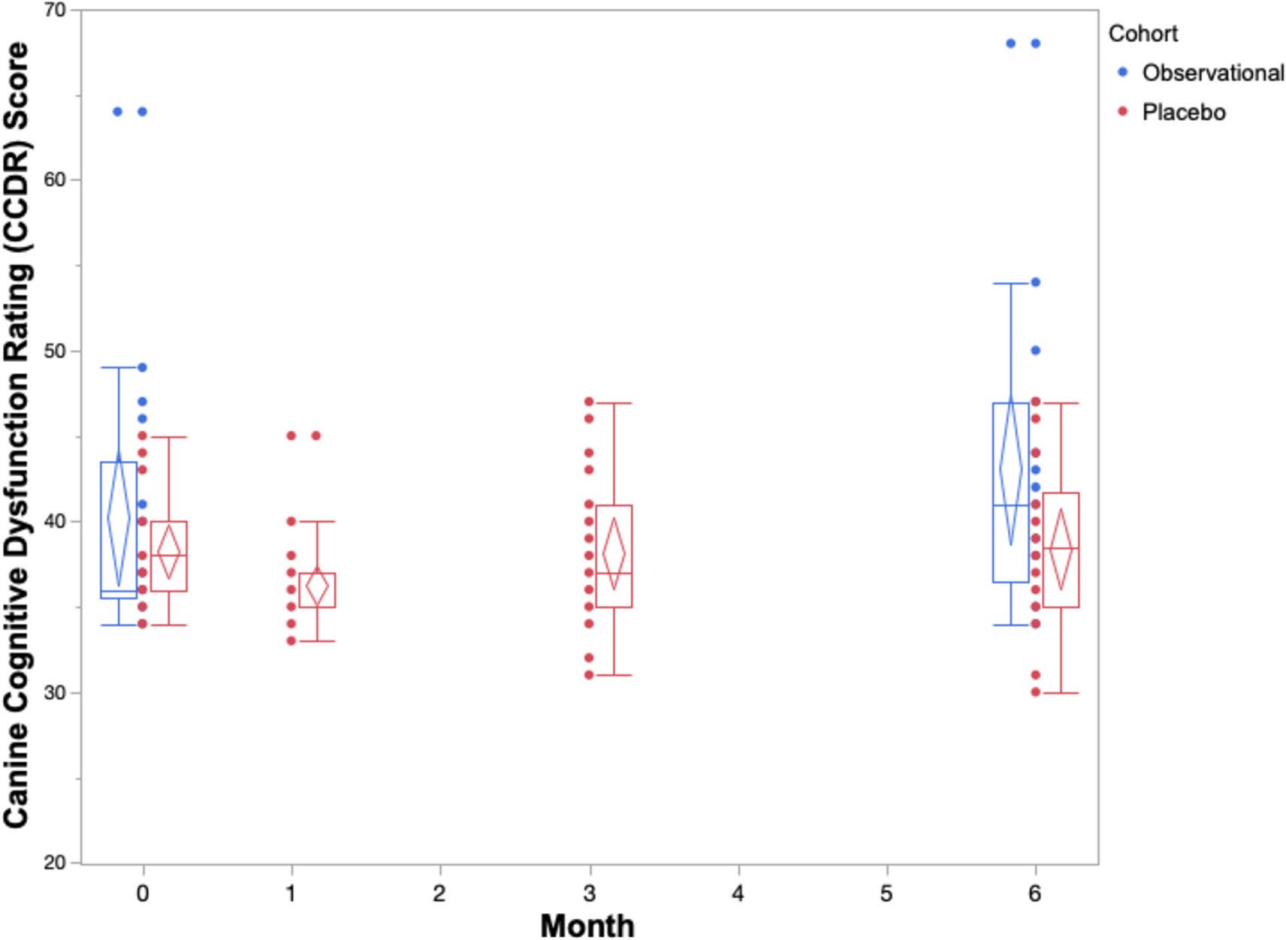
Box and whisker plots with confidence diamonds (95% confidence interval for the mean) of CCDR scores of two cohorts of elderly dogs across 6 months. Data in red represents dogs who were receiving a double-blinded placebo treatment as part of a randomized controlled trial. Data in blue represents dogs who were participating in an observational, longitudinal cohort study on the neurological changes of aging. Higher CCDR scores indicate increased cognitive impairment

**Fig. 2:**
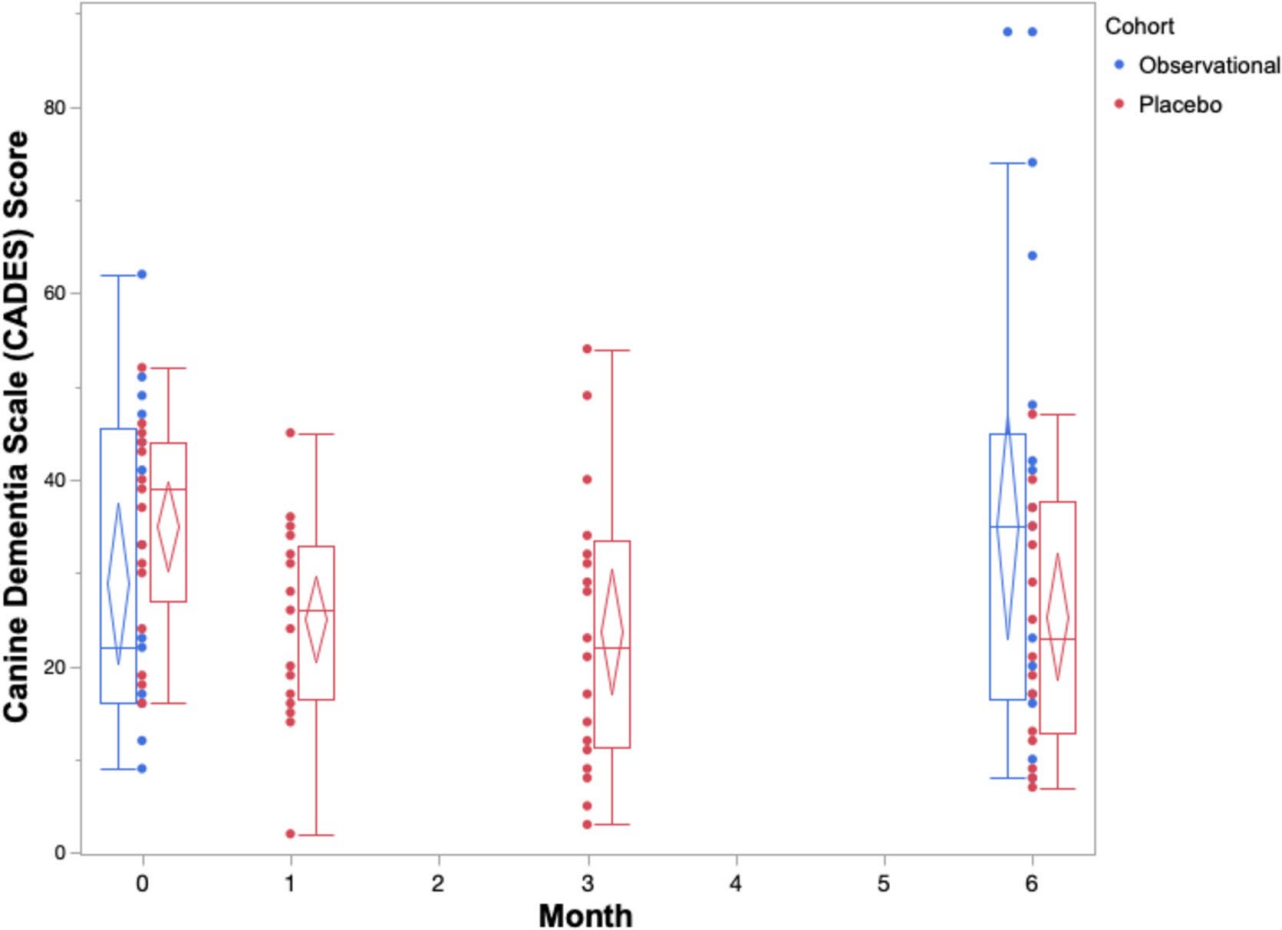
Box and whisker plots with confidence diamonds (95% confidence interval for the mean) of CADES scores of two cohorts of elderly dogs across 6 months. Data in red represents dogs who were receiving a double-blinded placebo treatment as part of a randomized controlled trial. Data in blue represents dogs who were participating in an observational, longitudinal cohort study on the neurological changes of aging. Higher CADES scores indicate increased cognitive impairment

**Fig. 3:**
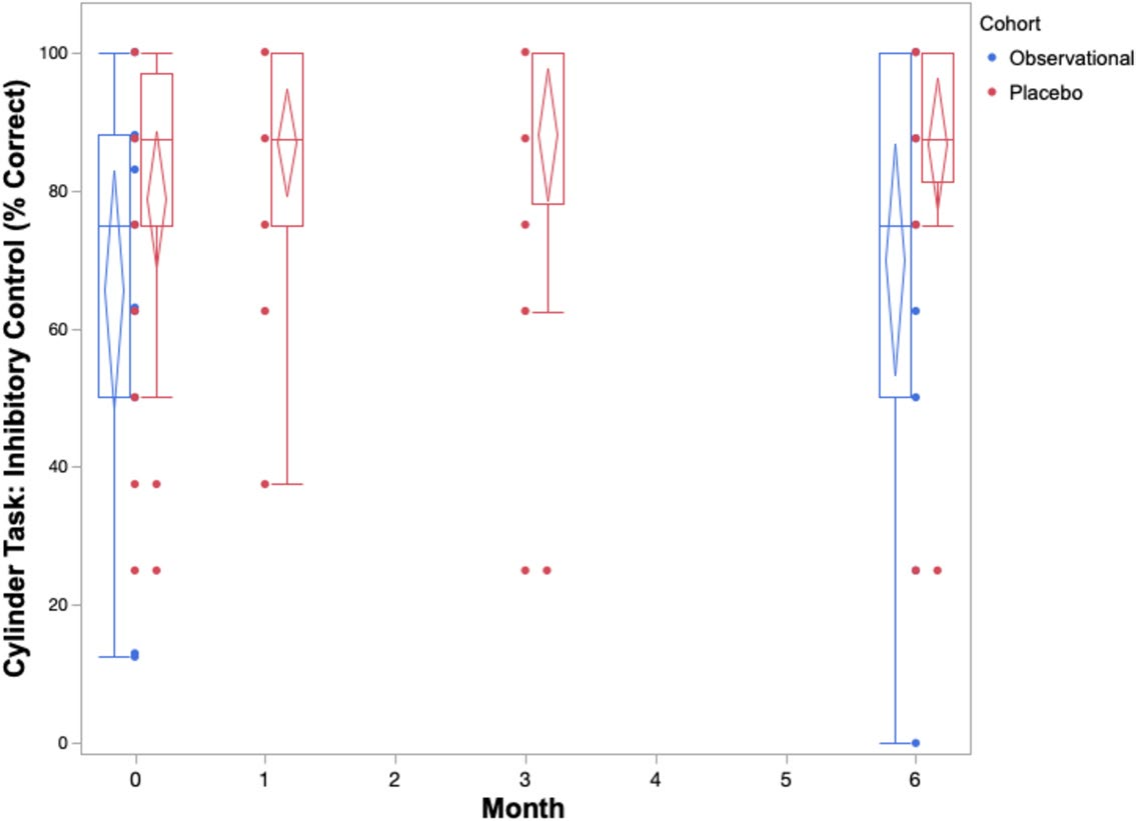
Box and whisker plots with confidence diamonds (95% confidence interval for the mean) of inhibitory control scores (% correct out of 8 trials) in two cohorts of elderly dogs across 6 months. Data in red represents dogs who were receiving a double-blinded placebo treatment as part of a randomized controlled trial. Data in blue represents dogs who were participating in an observational, longitudinal cohort study on the neurological changes of aging. Lower inhibitory control scores indicate increased cognitive impairment

**Fig. 4:**
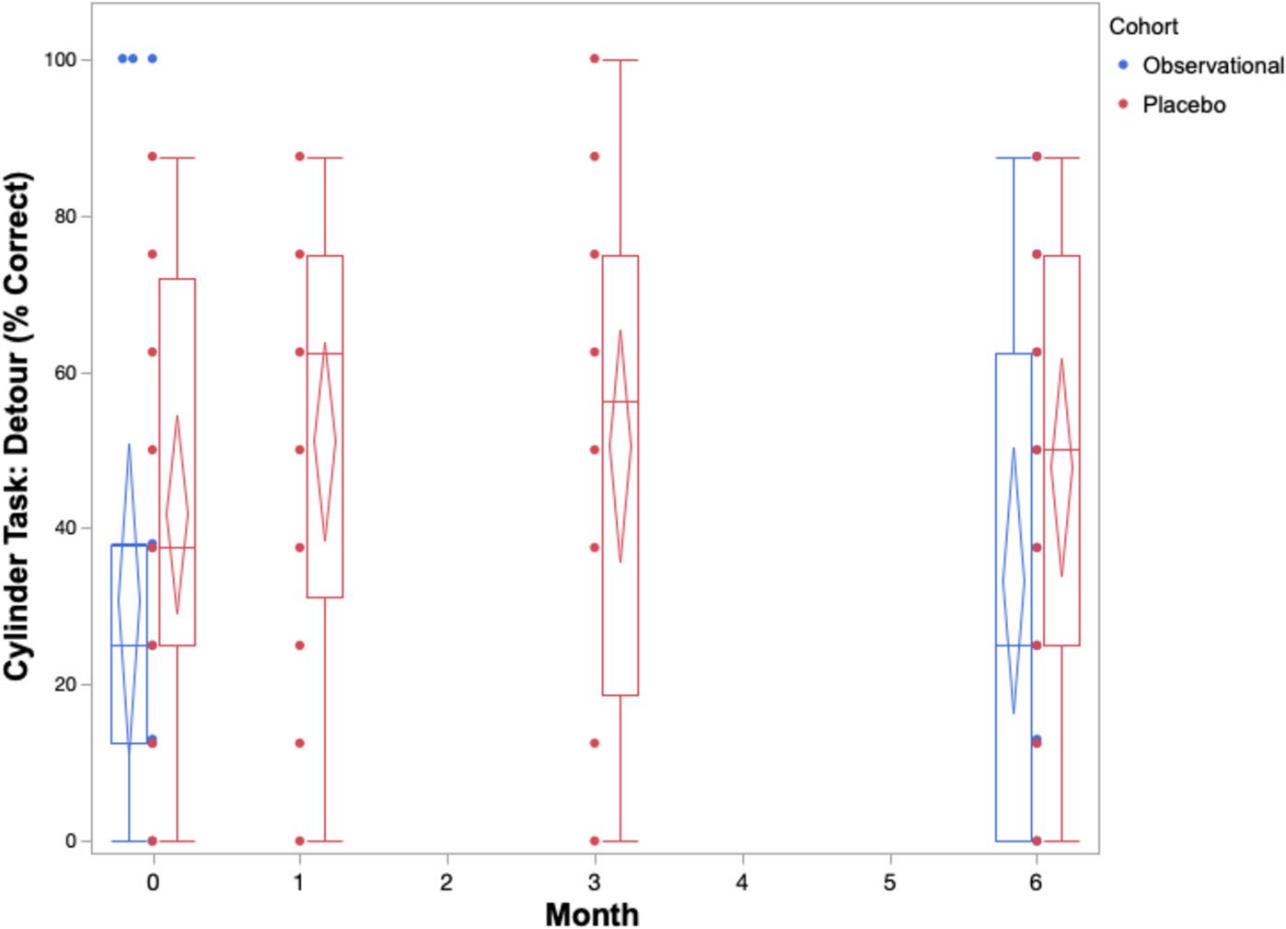
Box and whisker plots with confidence diamonds (95% confidence interval for the mean) of detour scores (% correct out of 8 trials) in two cohorts of elderly dogs across 6 months. Data in red represents dogs who were receiving a double-blinded placebo treatment as part of a randomized controlled trial. Data in blue represents dogs who were participating in an observational, longitudinal cohort study on the neurological changes of aging. Lower detour scores indicate increased cognitive impairment

**Fig. 5:**
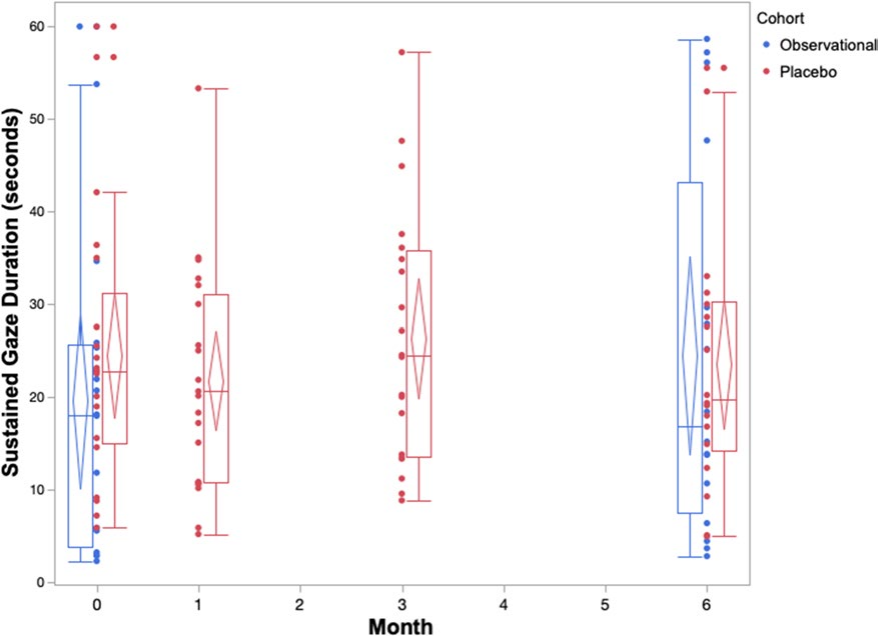
Box and whisker plots with confidence diamonds (95% confidence interval for the mean) of sustained gaze duration (average seconds) in two cohorts of elderly dogs across 6 months. Data in red represents dogs who were receiving a double-blinded placebo treatment as part of a randomized controlled trial. Data in blue represents dogs who were participating in an observational, longitudinal cohort study on the neurological changes of aging. Lower sustained gaze duration indicates increased cognitive impairment

The cognitive performance of the placebo cohort across 6 months on both owner assessments and in-house assessments is summarized in Table 2 and Figs. 1, 2, 3, 4, and 5. The reduced sample size (*n* = 20) in the cylinder tasks at baseline is attributed to one dog who would not obtain treats consistently from inside the cylinder. The same dog later adapted to the task and successfully completed them in all subsequent visits. Over the course of the study, one dog in the placebo cohort passed away before the 3-month visit. By 6 months, a second dog passed away, one withdrew from the study, and another’s mobility deteriorated such that it could not perform the cylinder tasks.

**Table 2:** Matched pairs and effect size between performance at baseline and three end points (1 month, 3 months, 6 months) in a placebo cohort of senior dogs

	Baseline (0 month) score	“End” point	“End” point score	*p* value	Effect size (Hedge’s *g*_av_)
Canine Cognitive Dysfunction Rating (CCDR) score^△^	Mean: 38.29 SD: 3.49 *(n* = *21)*	1 month *(n* = *21)*	Mean: 36.29 SD: 2.57	0.01	*g* = 0.65
		3 months *(n* = *20)*	Mean: 38.15 SD: 4.42	0.81	*g* = 0.05
		6 months *(n* = *18)*	Mean: 38.39 SD: 4.77	0.81	*g* = 0.009
Canine Dementia Scale (CADES) score^△^	Mean: 35 SD: 10.65 *(n* = *21)*	1 month *(n* = *21)*	Mean: 25.14 SD: 10.05	< 0.0001	*g* = 0.93
		3 months *(n* = *20)*	Mean: 23.75 SD: 14.32	< 0.0001	*g* = 0.86
		6 months *(n* = *18)*	Mean: 25.33 SD: 13.51	0.02	*g* = 0.76
Cylinder task: inhibitory control performance (% correct)^⬦^	Mean: 78.75 SD: 21.11 *(n* = *20)*	1 month *(n* = *21)*	Mean: 86.90 SD: 16.99	0.10	*g* = 0.42
		3 months *(n* = *20)*	Mean: 88.13 SD: 20.47	0.07	*g* = 0.43
		6 months *(n* = *17)*	Mean: 86.76 SD: 18.47	0.10	*g* = 0.38
Cylinder task: detour performance (% correct)^⬦^	Mean: 41.88 SD: 26.99 *(n* = *20)*	1 month *(n* = *21)*	Mean: 51.19 SD: 27.64	0.18	*g* = 0.33
		3 months *(n* = *20)*	Mean: 50.63 SD: 31.54	0.20	*g* = 0.33
		6 months *(n* = *17)*	Mean: 47.79 SD: 26.97	0.39	*g* = 0.25
Sustained gaze duration (seconds)^⬦^	Mean: 24.53 SD: 14.68 *(n* = *21)*	1 month *(n* = *21)*	Mean: 21.8 SD: 11.76	0.35	*g* = 0.20
		3 months *(n* = *20)*	Mean: 26.34 SD: 13.69	0.63	*g* = 0.13
		6 months *(n* = *18)*	Mean: 19.27 SD:14.00	0.89	*g* = 0.34

^△^Higher score indicates deterioration, lower score indicates improvement; ^⬦^Higher score indicates improvement; lower score indicates deterioration; *SD*, standard deviation

The box and whiskers plots demonstrate an observable difference in range across the five outcomes. The CCDR scores span a much smaller range when compared to the CADES scores across all timepoints. The three in-house assessments exhibit extreme individual heterogeneity, with large ranges across the 6 months. When considering the change in median, range, and confidence diamonds over 6 months, the CCDR, inhibitory control performance, and sustained gaze duration remain stable (Figs. 1, 3, 5). By contrast, both the CADES and detour task scores of the placebo cohort improved after 1 month and maintained that improvement to 6 months (Figs. 2, 4).

The results of the matched pairs analysis and effect size calculations on all outcome measures for the placebo cohort are provided in Table 2. Using the CCDR assessment, dogs in the placebo cohort showed significant improvement (a decrease in score) after 1 month (*p* = 0.01). However, scores stabilized after 3 and 6 months, showing no significant difference from baseline (*p* = 0.81, *p* = 0.81). The magnitude (effect size) of improvement (placebo effect) after 1 month is medium (*g* = 0.65) however, this wanes considerably over time, as indicated by negligible effect sizes after three and 6 months (*g*_av_ = 0.05 and *g*_av_ = 0.009, respectively). This contrasts the results observed with the CADES questionnaire. There is a significant improvement (decrease in score) in CADES scores at each of the three time points of the RCT when compared to baseline: 1 month (*p* < 0.0001), 3 months (*p* < 0.0001), and 6 months (*p* = 0.02). These lasting placebo effects are not only significant, but they are also substantial, as indicated by large effect sizes after 1 month and 3 months (*g*_av_ = 0.93 and *g*_av_ = 0.86, respectively) and medium effect size after 6 months (*g*_av_ = 0.76).

On all three of our in-house assessments (inhibitory control, detour, and sustained gaze), the placebo cohort experienced no significant changes (either improvements or deteriorations) over 6 months. Furthermore, these changes were small in magnitude, as reflected in the small effect sizes on both cylinder tasks ranging from *g*_av_ = 0.25 to *g*_av_ = 0.43 across all timepoints. Similarly, the effect size for sustained gaze was considered negligible at the 1-month (*g*_av_ = 0.18) and 3-month (*g*_av_ = 0.13) timepoints, and small at the 6-month timepoint (*g*_av_ = 0.34).

### Observational study cohort performance over 6 months

The baseline and second visit (6 months after baseline) data for the observational cohort are summarized in Table 3 and Figs. 1, 2, 3, 4, and 5. Reduced sample sizes across the three in-house assessments resulted from varying participation in the tasks. One dog refused all three tasks at both baseline and 6-month visits. Two dogs disengaged from the cylinder task after the inhibitory control and therefore did not have a detour score at baseline. One of these dogs was able to adapt and participate across all assessments at the 6-month visit, while the other deteriorated such that it would not participate in either cylinder task. Another dog would not engage with the cylinder at baseline or any in-house tasks at the 6-month visit.

**Table 3:** Matched pairs and effect size between baseline and 6-month performance of an observational cohort of senior dogs

	Baseline (0 month) score	6-month score	*p* value	Effect size (Hedge’s *g*_av_)
Canine Cognitive Dysfunction Rating (CCDR) score^△^	Mean: 40.24 SD: 7.68 *(n* = *17)*	Mean: 43.12 SD: 8.56 *(n* = *17)*	0.03	*g* = 0.35
Canine Dementia Scale (CADES) score^△^	Mean: 28.94 SD: 16.67 *(n* = *17)*	Mean: 35.06 SD: 23.40 *(n* = *17)*	0.07	*g* = 0.30
Cylinder task: inhibitory control performance (% correct)^⬦^	Mean: 65.63 SD: 31.27 *(n* = *15)*	Mean: 70 SD: 30.18 *(n* = *15)*	0.74	*g* = 0.14
Cylinder task: detour performance (% correct)^⬦^	Mean: 30.85 SD: 32.91 *(n* = *13)*	Mean: 33.37 SD: 30.47 *(n* = *15)*	0.78	*g* = 0.08
Sustained gaze duration (seconds)^⬦^	Mean: 19.59 SD: 17.65 *(n* = *16)*	Mean: 24.50 SD: 19.96 *(n* = *16)*	0.23	*g* = 0.25

^△^Higher score indicates deterioration, lower score indicates improvement; ^⬦^Higher score indicates improvement; lower score indicates deterioration; *SD*, standard deviation

The box and whiskers plots show a larger range across all five cognitive outcomes scores in the observational cohort compared to the placebo cohort (Figs. 1, 2, 3, 4, and 5).

The results of the matched pairs analysis and effect size calculations on all outcome measures for the observational cohort are provided in Table 3. Contrary to results observed in the placebo cohort, there was a small (*g*_av_ = 0.35), but significant (*p* = 0.03) deterioration with the CCDR (increase in score) after 6 months in the observational cohort. While not significant (*p* = 0.07), a similar, small-sized deterioration (*g*_av_ = 0.30) is observed when owners assessed their dog using CADES.

The observational cohort showed similar results to the placebo group on the in-house cognitive assessments, with no significant changes across 6 months. Effect sizes were comparable to the placebo cohort: negligible for both inhibitory control (*g*_av_ = 0.14) and detour (*g*_av_ = 0.08), and small with sustained gaze (*g*_av_ = 0.25).

### Comparison of cognitive performance between cohorts

A summary of the changes in score after 6 months in both the observational and placebo cohorts are reported in Table 4. Statistically, there was a medium (*g*_s_ = 0.51), but non-significant (*p* = 0.13) difference between the cohorts’ change in CCDR score after 6 months. This is different to the CADES owner assessment which showed a large (*g*_s_ = 1.08) and significant (*p* = 0.003) difference between the groups. The observational cohort experienced an average increase in CADES score (deterioration) whereas the placebo cohort experienced an average decrease in score (improvement). When directly comparing performance on the three in-house assessments between cohorts, there were small, non-significant differences in inhibitory control (*g*_s_ = 0.30), *p* = 0.75), detour (*g*_s_ = 0.14, *p* = 0.71), and sustained gaze (*g*_s_ = 0.29, *p* = 0.40) scores.

**Table 4:** Change in cognitive outcome measures score (month 6–baseline) by cohort

	Placebo cohort	Observational cohort	*p* value	Effect size (Hedge’s *g*_s_)
Change in Canine Cognitive Dysfunction Rating (CCDR) score^⬦^	Mean: 0.28 SD: 4.93 *(n* = *18)*	Mean: 2.88 SD: 4.95 *(n* = *17)*	0.13	*g* = 0.51
Change in Canine Dementia Scale (CADES) score^⬦^	Mean: − 8.44 SD: 13.40 *(n* = *18)*	Mean: 6.11 SD: 13.00 *(n* = *17)*	0.003	*g* = 1.08
Change in cylinder task: inhibitory control performance (% correct) ^△^	Mean: 8.59 SD: 19.75 *(n* = *16)*	Mean: 2.04 SD: 22.85 *(n* = *14)*	0.41	*g* = 0.30
Change in cylinder task: detour performance (% correct)^△^	Mean: 7.81 SD: 35.02 *(n* = *16)*	Mean: 2.85 SD: 35.68 *(n* = *13)*	0.71	*g* = 0.14
Change in sustained gaze duration (seconds)^△^	Mean: − 0.61 SD: 18.82 *(n* = *18)*	Mean: 4.16 SD: 12.95 *(n* = *15)*	0.40	*g* = 0.29

^△^Positive change indicates improvement; negative change indicates deterioration

^⬦^Negative change indicates improvement; positive change indicates deterioration

*SD*, standard deviation

Table 5 displays the parameters from each cognitive assessment’s mixed effects model. In accordance with the matched pairs analysis, the only model to show a significant time × cohort effect (*p* = 0.03) was in the CADES model. The models of CCDR, inhibitory control, detour, and sustained gaze all showed non-significant time × cohort effects (*p* = 0.20, 0.59, 0.95, and 0.41 respectively) after controlling for the effects of age, weight, and sex. Therefore, both the *T-*tests and mixed effects models demonstrate a significant difference in CADES score between cohorts over time.

**Table 5:** Parameters from mixed effects models of each cognitive assessment

Response variable	Model effects	Estimate (β)	SE	t ratio	*p* value	95% CI
Canine Cognitive Dysfunction Rating (CCDR) score	Cohort × month	0.30	0.23	1.30	0.20	− 0.16, 0.77
	Age	1.62	0.59	2.74	0.01*	0.42, 2.82
	Weight	0.02	0.10	0.20	0.85	− 0.18, 0.21
	Sex	− 0.56	1.06	− 0.53	0.60	− 2.72, 1.60
	Cohort	3.56	1..64	2.16	0.04*	0.25, 6.87
	Month	0.18	0.16	1.14	0.26	− 0.13, 0.49
Canine Dementia Scale (CADES) scores	Cohort × month	2.09	0.72	2.9	0.03*	0.66, 3.53
	Age	5.08	1.51	3.37	0.002*	2.00, 8.16
	Weight	0.3	0.24	1.21	0.24	− 0.20, 0.79
	Sex	− 1.27	2.69	− 0.47	0.64	− 6.77, 4.23
	Cohort	1.92	4.33	0.44	0.66	− 6.81, 10.65
	Month	− 1.08	0.48	− 2.25	0.03	− 2.03, − 0.12
Cylinder task: inhibitory control performance (% correct)	Cohort × month	− 0.60	1.12	− 0.54	0.59	− 2.83, 1.63
	Age	− 6.51	2.41	− 2.70	0.01*	− 11.42, − 1.60
	Weight	0.23	0.39	0.60	0.55	− 0.56, 1.03
	Sex	− 3.26	4.24	− 0.77	0.45	− 11.95, 5.43
	Cohort	− 18.68	6.80	− 2.75	0.009*	− 32.39, − 4.97
	Month	0.99	0.72	1.38	0.17	− 0.44, 2.42
Cylinder task: detour performance (% correct)	Cohort × month	− 0.13	1.93	− 0.06	0.95	− 3.98, 3.73
	Age	− 7.41	2.61	− 2.84	0.008*	− 12.75, − 2.07
	Weight	− 0.38	0.40	− 0.93	0.36	− 1.21, 0.45
	Sex	− 2.69	4.31	− 0.62	0.54	− 11.54, 6.17
	Cohort	− 17.74	7.87	− 2.25	0.03	− 33.65, − 1.83
	Month	0.50	1.22	0.41	0.69	− 1.94, 2.93
Sustained gaze duration (seconds)	Cohort × month	0.70	0.84	0.83	0.41	− 0.97, 2.36
	Age	− 2.82	1.42	− 1.98	0.06	− 5.73, 0.09
	Weight	0.27	0.23	0.25	0.25	− 0.20, 0.75
	Sex	3.39	2.52	0.19	0.19	− 1.77, 8.55
	Cohort	1.54	4.22	0.36	0.72	− 6.99, 10.06
	Month	0.05	0.54	0.08	0.93	− 1.03, 1.12

*SE*, standard error

*CI*, confidence interval

### Owner-reported changes in cognitive status categories across 6 months

Individual clinical severities are reported in Table 6, categorized based on CCDR stratifications [32]. There was a similar distribution across categories in both cohorts, with most dogs classified as not affected. Only one dog in the observational cohort was classified as having cognitive dysfunction based on CCDR score at baseline. After 6 months, more dogs in the observational cohort deteriorated (*n* = 7) compared to the placebo cohort (*n* = 4).

**Table 6:** Canine Cognitive Dysfunction Rating (CCDR) clinical categories across 6 months by cohort

	Placebo cohort	Observational cohort
Baseline (0 month) status	Not affected: *n* = 14/21 (66.7%) At-risk: *n* = 7/21 (33.3%)	Not affected: *n* = 10/17 (58.8%) At-risk: *n* = 6/17 (35.3%) CCD: *n* = 1/17 (5.9%)
6-month status	Not affected: *n* = 12/18 (66.7%) At-risk: *n* = 6/18 (33.3%)	Not affected: *n* = 6/17 (35.3%) At-risk: *n* = 8/17 (47.1%) CCD: *n* = 3/17 (17.6%)
Change in severity category	Improvement (at-risk → not affected): *n* = 4/18 (22.2%) No change (not affected): *n* = 8/18 (44.4%) No change (at-risk): *n* = 2/18 (11.1%) Deterioration (not affected → at-risk): *n* = 4 (22.2%)	Improvement (at-risk → not affected): *n* = 1/17 (5.9%) No change (not affected): *n* = 5/17 (29.4%) No change (at-risk): *n* = 3/17 (17.6%) No change (CCD): *n* = 1/17 (5.9%) Deterioration (not affected → at-risk): *n* = 5/17 (29.4%) Deterioration (at-risk → CCD) *n* = 2/17 (11.8%)

*CCD*, Canine Cognitive Dysfunction

Clinical severities based on CADES classifications are described in Table 7 [31]. At baseline, most dogs (*n* = 14) in the placebo cohort were classified as moderately affected, whereas most (*n* = 10) dogs in the observational cohort were classified as mildly affected. After 6 months, eight dogs in the placebo cohort exhibited improvements in CADES severity category compared to only one dog improving from the observational cohort. However, the same number of dogs (*n* = 3) deteriorated across both cohorts.

**Table 7:** Canine Dementia Scale (CADES) clinical categories across 6 months by cohort

	Placebo cohort	Observational cohort
Baseline (0 month) status	mild: *n* = 4/21 (19.0%) moderate: *n* = 14/21 (66.7%) severe: *n* = 3/21 (14.3%)	mild: *n* = 10/17 (58.8%) moderate: *n* = 3/17 (17.6%) severe: *n* = 4/17 (23.5%)
6-month status	no impairment: *n* = 1/18 (5.6%) mild: *n* = 8/18 (44.4%) moderate *n* = 7/18 (38.9%) severe: *n* = 2/18 (11.1%)	mild: *n* = 8/17 (47.1%) moderate: *n* = 5/17 (29.4%) severe: *n* = 4/17 (23.5%)
Change in severity category	Improvement (moderate → no impairment): *n* = 1/18 (5.6%) Improvement (moderate → mild): *n* = 5/18 (27.8%) Improvement (severe → moderate): *n* = 2/18 (11.1%) No change (mild): *n* = 3/18 (16.7%) No change (moderate): *n* = 4/18 (22.2%) Deterioration (mild → moderate): *n* = 1/18 (5.6%) Deterioration (moderate → severe): *n* = 2/18 (11.1%)	Improvement (moderate → mild): *n* = 1/17 (5.9%) No change (mild): *n* = 7/17 (41.2%) No change (moderate): *n* = 2/17 (11.8%) No change (severe): *n* = 4/17 (23.5%) Deterioration (mild → moderate): *n* = 3/17 (17.6%)

## Discussion

The current landscape of clinical trials investigating aging and cognitive decline in dogs spans a wide range of outcome measures, durations, and study designs including RCTs and open label trials with and without controls [44–52]. This lack of uniformity makes it challenging to understand how outcome measures are performing in controlled settings and how the placebo effect contributes to their performance. In this paper, we examined the performance of elderly dogs across five cognitive outcome measures in both an observational and placebo-controlled setting. Our results demonstrate that different outcome measures vary in their susceptibility to placebo effect, with owner questionnaires exhibiting a stronger effect than in-house assessments. Additionally, the duration of placebo effect is outcome measure dependent, as evident by sustained improvements in CADES score, but not CCDR scores, through 6 months in a placebo cohort.

We observed that owners’ ratings of their dog’s cognitive performance were dependent on both the questionnaire used, as well as the study setting in which the questionnaire was administered. Dogs in the placebo cohort exhibited strong and significant cognitive improvements on the CADES assessment, which weres sustained through 1-, 3-, and 6-month timepoints. When using the CCDR assessment, the same group experienced a smaller, but still significant, improvement after 1 month, which then waned by 3 months. It is unlikely that a placebo treatment can improve cognitive status. Therefore, these reported improvements likely reflect a caregiver placebo response, which persists longer when using the CADES. This is further supported by the different trajectory observed in the observational study cohort. When owners were not prompted with the possibility of an active (or placebo) treatment, they reported a small and significant increase in CCDR score (indicating cognitive deterioration) after 6 months, and a small, non-significant increase in CADES score (also indicating cognitive deterioration). Because we did not include a control group who did not participate in any type of in-house evaluation, the natural trajectory of cognitive decline outside a trial setting remains unclear. However, other studies demonstrate a progressive deterioration in either CCDR or CADES similar to that observed in our observational study cohort [31, 53]. While both groups likely experienced some positive effects from engagement in the study setting (such as increased socialization or increased attention to health), the differences observed between the observational and placebo groups support the suggestion that placebo administration confers benefits which attenuate cognitive decline beyond that of trial participation alone. Moreover, given that the RCT involved an active treatment arm, it is likely that a selection bias occurred, favoring owners who were optimistic to see an improvement in their dog. In contrast, caregivers in the observational cohort may have been less influenced by expectations of change, as they enrolled in the study without the motivation of beginning a specific intervention. It would be difficult to disentangle the specific differences between trial participation as opposed to placebo effect as even the suggestion of placebo introduces expectancy. For similar reasons, isolating the effects of trial participation from the natural course of cognitive decline is challenging, as study involvement itself (regardless of how involved or invasive) may alter outcomes through increased attention, monitoring, or engagement (by either the caregiver or the examiner).

The difference between CCDR and CADES within the same group may be explained by differing language, sensitivity, structure, and recall periods. The CCDR was designed to distinguish dogs with severe, diagnosed dementia, whereas the CADES was developed to be sensitive to early and mild cognitive changes. As such, the CADES is likely more sensitive to subtle shifts in caregiver perception or expectancy effects. Furthermore, the optimal interval to administer these surveys has yet to be determined, making the distinct language differences and recall periods between CCDR and CADES better suited for different sampling intervals. While both CCDR and CADES use a 5-point scale, the response options differ. CCDR focuses on recent behavioral changes (no greater than a month prior) or allows owners to compare current behavior to 6 months previously. In contrast, CADES requires broader recall over a longer timeframe, asking owners to recall all behaviors over the past 6 months. Therefore, when used in the RCT, a portion of the recall period in the CADES falls outside of the trial period, such as with the 1- and 3-month time points. This may make it difficult for owners to respond objectively and could increase their susceptibility to placebo effects, which are already heightened in an RCT setting. Therefore, it is important to align assessment tools with study design, ensuring the cognitive measures accurately capture treatment effects within the intended timeframe.

The differing cognitive decline trajectories observed between cohorts may be explained by several factors, including the selection bias and caregiver optimism or expectancy effects as well as differences in assessment frequency. The RCT dogs experienced more frequent visits that each involved a physical exam and multiple in-house assessments, which may have introduced physical or cognitive effects that confounded outcomes (either positively or negatively). However, future studies are required to determine whether concurrent physical examination and cognitive testing has a significant effect on cognitive performance. Like their dogs, caregivers may have been impacted by the differing visit frequency between cohorts. Longer intervals between visits, as with the observational study, may reduce response fatigue. However, most studies examine response fatigue in lengthy single surveys rather than repeated longitudinal responses [54–56]. Increasing intervals may also decrease recall [57, 58], and in an elderly study population, it may increase risk of attrition [59–61]. Owners in the RCT were assessing their dogs at a higher frequency than those participating in the observational study. Increased assessment frequency, coupled with stricter compliance guidelines in RCTs, may heighten owners’ awareness of their pet’s behavior, potentially influencing their questionnaire responses. This phenomenon is not dissimilar to the “protocol effect” or the “care effect” described in human medicine [17, 62]. Furthermore, increased attentiveness may lead to modifications in owner-dog interactions, such as greater social engagement and mental stimulation, which could yield genuine cognitive and behavioral improvements in the dog[23]. This has been termed the veterinary “placebo-by-proxy effect,” wherein an owner’s expectations and behavioral changes result in actual benefits for the animal. However, it is difficult to say whether such an effect occurred across the RCT, as there were no improvements observed in the in-house cognitive assessments. If a true placebo-by-proxy effect had taken place, improvements might have been expected across all measures rather than being limited to owner-reported questionnaires.

The in-house cognitive assessments are more objective performance evaluations. Across both studies, performance on the in-house cognitive assessments remained stable over 6 months. Unlike the questionnaires, these tests may be susceptible to other biases in addition to the placebo effect, such as practice effect (when a subject improves on a task due to repeated performances) [63, 64] or familiarity effect (when a subject becomes more comfortable with the test environment or experimenter and improves on a task) [65, 66]. However, we did not observe a practice effect as the placebo cohort did not demonstrate a significant improvement over the observational cohort in any of the three outcomes, despite increased testing repetitions at 1 and 3 months. Some dogs may have experienced the benefits of familiarity effect, particularly those who would not engage with one or all three tests at baseline but then would participate in subsequent visits. However, this could also be due to waxing and waning of clinical signs or daily mood changes. It is clear more work is necessary on the longitudinal performance (beyond 6 months) of these outcome measures to determine clinically meaningful changes. However, the stability in performance across both cohorts supports that these in-house assessments, more than the owner assessments, are resistant to many confounding effects.

Our study has some limitations. The first is that the owner questionnaires were administered in an online format. The CADES questionnaire specifically is designed to be administered by and discussed with a veterinarian, so that other comorbidities which may contribute to behavioral changes can be ruled out. However, owners received detailed training on questionnaires at enrollment of the study. In addition, dogs underwent physical examinations and blood work at each time point to monitor for the emergence of new comorbidities. Another limitation is our sample size was relatively small and only spanned a 6-month period. Future work should be done in a larger population, across a longer time span to understand how and when individuals decline based on these cognitive assessments.

Together, our findings demonstrate that owner assessments of canine cognition administered online in a clinical trial setting are susceptible to placebo effect, while in-house cognitive assessments were more resistant. The placebo effect in CCDR is small and short-lived, while changes in CADES scores are larger and persist for at least 6 months. The observed trajectory differences in outcome measures across the same populations over the same duration emphasize the importance of using multiple cognitive assessments. The differences between our observational and placebo cohorts further demonstrate the need for thoughtful selection of outcomes which align with study design. Indeed, cognition is complex, and differing assessments may capture different cognitive behaviors or domains. This study provides critical insight into the magnitude and duration of the placebo effect when using five different canine cognitive outcome measures. Applying this understanding of the placebo effect will allow for the design of more effective and rigorous veterinary clinical trials on canine cognitive decline.

## Supplementary Information

Below is the link to the electronic supplementary material.

Supplementary file1 (XLSX 15 KB)

## Data Availability

All data analyzed are provided as a supplementary file (Supplementary data 1).
